# Craniocervical instability in patients with Ehlers-Danlos syndromes: outcomes analysis following occipito-cervical fusion

**DOI:** 10.1007/s10143-023-02249-0

**Published:** 2024-01-02

**Authors:** Fraser C. Henderson, Jane R. Schubart, Malini V. Narayanan, Kelly Tuchman, Susan E. Mills, Dorothy J. Poppe, Myles B. Koby, Peter C. Rowe, Clair A. Francomano

**Affiliations:** 1 Department of Neurosurgery, University of Maryland School of Medicine, Baltimore, MD USA; 2 The Metropolitan Neurosurgery Group LLC, Silver Spring, MD USA; 3 Department of Surgery, Penn State College of Medicine, Hershey, PA USA; 4 Division of Neurosurgery, University of Maryland Capital Region Medical Center, Largo, Maryland USA; 5 Bobby Jones Chiari & Syringomyelia Foundation, Staten Island, New York USA; 6 Luminis Health, Doctors Community Medical Center, Lanham, Maryland USA; 7 Department of Pediatrics, Johns Hopkins University School of Medicine, Baltimore, MD USA; 8 Department of Medical and Molecular Genetics, Indiana University School of Medicine, Indianapolis, Indiana USA

**Keywords:** Occipito-cervical fusion, Basion axis interval, Horizontal Harris measurement, Ventral brainstem compression, Clivo-axial angle, Cervical medullary syndrome

## Abstract

Craniocervical instability (CCI) is increasingly recognized in hereditary disorders of connective tissue and in some patients following suboccipital decompression for Chiari malformation (CMI) or low-lying cerebellar tonsils (LLCT). CCI is characterized by severe headache and neck pain, cervical medullary syndrome, lower cranial nerve deficits, myelopathy, and radiological metrics, for which occipital cervical fusion (OCF) has been advocated. We conducted a retrospective analysis of patients with CCI and Ehlers-Danlos syndrome (EDS) to determine whether the surgical outcomes supported the criteria by which patients were selected for OCF. Fifty-three consecutive subjects diagnosed with EDS, who presented with severe head and neck pain, lower cranial nerve deficits, cervical medullary syndrome, myelopathy, and radiologic findings of CCI, underwent open reduction, stabilization, and OCF. Thirty-two of these patients underwent suboccipital decompression for obstruction of cerebral spinal fluid flow. Questionnaire data and clinical findings were abstracted by a research nurse. Follow-up questionnaires were administered at 5–28 months (mean 15.1). The study group demonstrated significant improvement in headache and neck pain (*p* < 0.001), decreased use of pain medication (*p* < 0.0001), and improved Karnofsky Performance Status score (*p* < 0.001). Statistically significant improvement was also demonstrated for nausea, syncope (*p* < 0.001), speech difficulties, concentration, vertigo, dizziness, numbness, arm weakness, and fatigue (*p* = 0.001). The mental fatigue score and orthostatic grading score were improved (*p* < 0.01). There was no difference in pain improvement between patients with CMI/LLCT and those without. This outcomes analysis of patients with disabling CCI in the setting of EDS demonstrated significant benefits of OCF. The results support the reasonableness of the selection criteria for OCF. We advocate for a multi-center, prospective clinical trial of OCF in this population.

**Supplementary information:**

The online version contains supplementary material available at 10.1007/s10143-023-02249-0.

## Introduction

The unique range of motion of the craniocervical junction relies upon the competence of ligaments joining the cranium to the upper two cervical vertebrae. Craniocervical instability (CCI) occurs in conditions of weakened ligaments such as trauma, infection, and connective tissue disorders. Inflammatory disorders, including rheumatoid arthritis and lupus, can also result in cranial settling and basilar invagination. Craniocervical instability and its phenotypic expression, the cervical medullary syndrome, have been increasingly recognized in conditions associated with ligamentous laxity. The latter include genetic conditions such as Down syndrome, congenital conditions such as Goldenhar syndrome, and hereditary disorders of connective tissue (HDCT), such as osteogenesis imperfecta, Marfan, Morquio, Stickler, and the Ehlers-Danlos syndromes [1–5]. Moreover, there is a recognized convergence of connective tissue disorders and “complex Chiari,” characterized by basilar invagination, kyphotic clival axial angle (CXA), and craniocervical instability [6–10]. This association has prompted increased consideration of dynamic imaging to better characterize the pathology and determine whether occipito-cervical fusion (OCF) may be indicated [11–18]. Emblematic of the HDCT are the 13 types of Ehlers-Danlos syndrome (EDS), characterized by weakness of connective tissue and many comorbid conditions, including neurological findings and dysautonomia attributed in part to chronic craniocervical and spinal instability [2, 19].

A growing body of literature suggests that chronic CCI manifests as a broad array of deleterious biomechanical effects upon the neural axis, in addition to causing altered cerebrospinal fluid and vascular flow [4, 8, 9, 12, 15–17, 20]. Headaches, long tract findings, motor delay and quadriparesis, dyspraxia, gait instability, and altered autonomic function are recognized as consequences of chronic biomechanical deformation of structures at the craniocervical junction in many hereditary connective tissue disorders [8, 13, 16, 19, 21–25]. There has been an evolving consensus in the literature of radiological metrics by which the presence, severity, and specific characteristics of CCI can be assessed and addressed [6, 8, 9, 13, 26–36].

Concurrent with this emerging understanding of chronic CCI is a need to validate clinical and radiological criteria by which individuals may be identified as appropriate candidates for OCF. This report describes a retrospective outcomes analysis of a cohort of patients with EDS and CCI who underwent OCF for severe, chronic, debilitating pain; symptoms of the cervical medullary syndrome; increasing neurological deficits; confirmatory radiological findings; and failed non-operative management. Our goal was to evaluate whether the surgical outcomes support the criteria by which patients were diagnosed with craniocervical instability and selected for OCF.

## Methods

The study population consisted of a consecutive series of adults (*n* = 53) diagnosed with EDS [25] with clinical and radiological findings of CCI.

### Perioperative data

All participants completed a clinical intake questionnaire on their initial visit, grading severity of pain, lightheadedness**,** syncope and presyncope, fatigue, mental clarity, and symptoms that constitute the cervical medullary syndrome [26]. Neurological examinations were performed by the neurosurgeons. Data were also extracted from clinical notes and other routine intake questionnaires.

### Radiological findings of instability

Patients underwent dynamic MRI and CT imaging where possible. In some cases, flexion–extension X-rays were performed. Radiological measurements were performed by the neuroradiologist (MK).

**Fig. 1:**
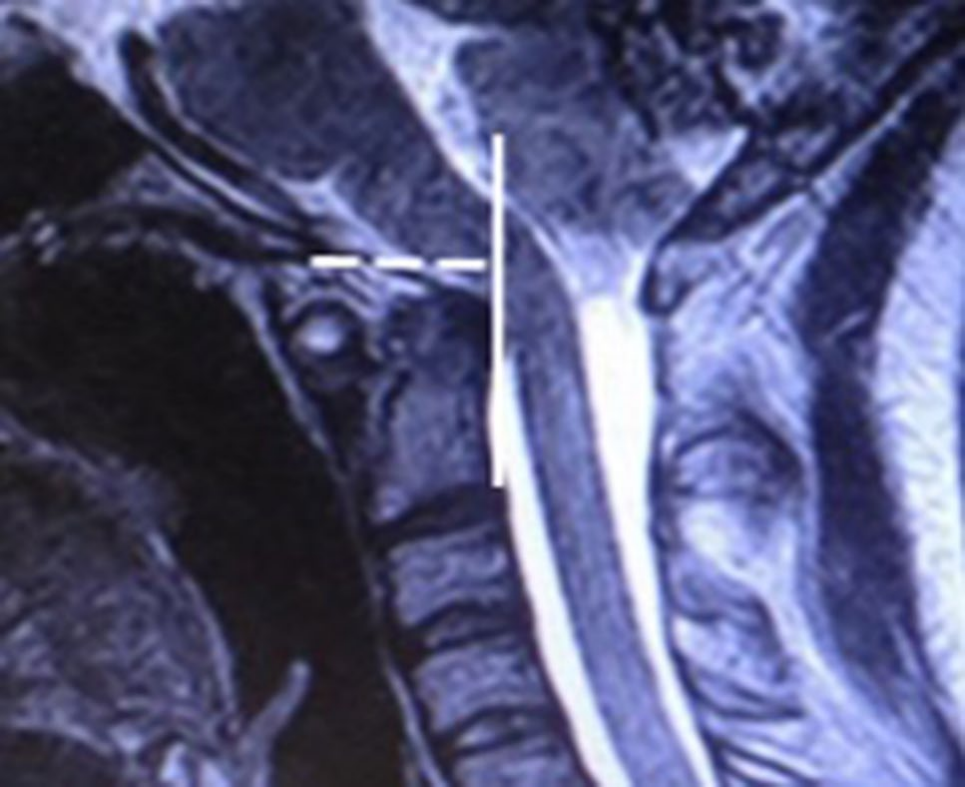
MRI, cervical spine, mid-sagittal neutral view, T2 weighted (1.5 Tesla), showing a basion axis interval (white dashed line), measured from the posterior axial line (solid white line) to the basion. The BAI measures 15 mm. This exceeds the pathological threshold of 12 mm and constitutes radiological evidence of CCI

1. Dynamic upright flexion–extension cervical spine MRI

- Horizontal Harris Measurement (HHM)–the *basion axis interval* (BAI). Abnormal is ≥ 12 mm [13, 26, 30, 32, 34, 37] (Fig. 1).

**Fig. 2:**
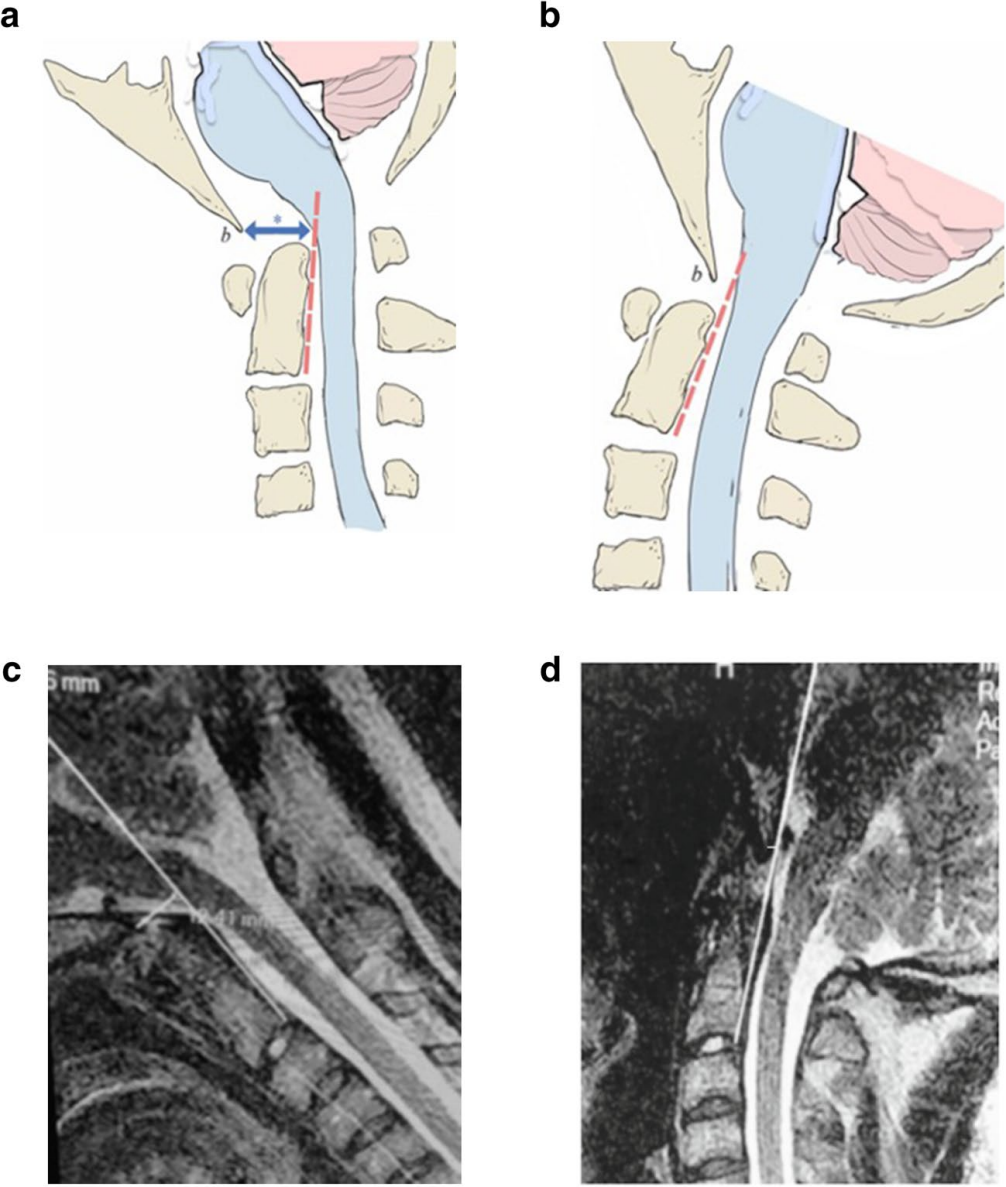
**a** Pathological translation of the basion with respect to the odontoid. Normally, between flexion and extension, the basion (b) pivots over the odontoid with < 2 mm of translation. In Fig. 2a, the cervical spine is in flexion, and the basion has translated anteriorly causing a bend in the brainstem. Note the BAI (*)—the interval measured from the basion to the posterior axial line (dashed line) is greater than the width of the spinal cord. **b** The cervical spine in extension shows straightening of the brainstem and a upper spinal cord and a shortened BAI. The change in BAI represents a pathological translation of the basion with respect to the spine. **c** MRI, upright, (dynamic), mid-sagittal, flexion view, T2 weighted (0.6 Tesla, Fonar Corp). The basion axis interval is 12 mm. **d** MRI, upright, (dynamic), mid-sagittal, extension view, T2 weighted (0.6 Tesla, Fonar Corp). The basion axis interval is 5 mm. Therefore, the BAI in flexion (12 mm) minus the BAI in extension (5 mm) represents a pathological translation of 7 mm

b. BAI translation between flexion and extension (BAI_flexion_–BAI_extension_). Abnormal is ∆BAI > 4 mm [13, 23, 26, 29, 33, 34, 36, 38] (Fig. 2a, b, c, d).

c. Clival axial angle (CXA). Abnormal is < 135° [9, 11, 26, 34, 39, 40].
d. Ventral brainstem compression as measured by pBC2 measurement (also known as Grabb-Mapstone-Oakes (GMO) or Grabb-Oakes measurement). Abnormal is pBC2 ≥ 9 mm [27, 28, 34].
e. MRI of cervical spine or brain to rule out Chiari malformation (CMI) (tonsillar herniation ≥ 5 mm), or low-lying cerebellar tonsils (LLCT) (tonsillar herniation < 5 mm), or foramen magnum (FM) stenosis [34, 41].

2. Dynamic supine CT of the cervical spine with full neck rotation to left and to right to assess atlantoaxial instability (AAI) [8, 19, 29, 31, 32, 34, 38], measured by one of the following: C1C2 angular displacement ≥ 41°, or lateral displacement C1 upon C2 ≥ 4 mm on lateral head tilt, or > 80% loss of facet overlap on 3D CT reconstruction.

### Indications for surgery

Patients undergoing surgery met each of these criteria (see Surgical Algorithm for the Treatment of Craniocervical Instability in the Ehlers Danlos Syndrome and Hypermobility Spectrum Disorder Populations Supplement):

1. Severe head and/or neck pain (≥ 7/10 on the visual analog scale) for > 6 months.
2. Symptoms of the cervical medullary syndrome: altered vision, diplopia, nystagmus, decreased hearing, dizziness, imbalance, vertigo, weakness, sensory loss, choking, dysarthria, dysphagia, sleep apnea or disordered sleep architecture, syncope, presyncope, and other dysautonomic symptoms [13, 19, 26, 42].
3. Neurological deficits congruent with craniocervical instability such as lower cranial nerve deficits, weakness, sensory changes, hyperreflexia, Hoffman reflex, absent abdominal reflexes, Romberg sign, abnormal tandem gait, and dysdiadochokinesia.
4. Failed non-operative management (neck brace, physical therapy, isometric exercises of the neck, activity modification, pain medication, and other modalities).
5. Radiological findings of CCI as described above and one or more of the following additional radiological findings: (i) CMI, LLCT, or FM stenosis causing CSF flow obstruction; (ii) kyphotic clival axial angle; (iii) AAI; (iv) ventral brainstem compression (pBC2 ≥ 9 mm).
6. Ability of the patient to understand the procedure, the risks and alternatives to surgery, and consent for surgery.

### Exclusion criteria for surgery

Patients were excluded from surgery if less than 17 years of age, if they had undergone a previous craniocervical or atlantoaxial fusion, if they were pregnant, or if they were experiencing severe medical complications requiring ongoing treatment elsewhere.

#### The surgical procedure

**Fig. 3:**
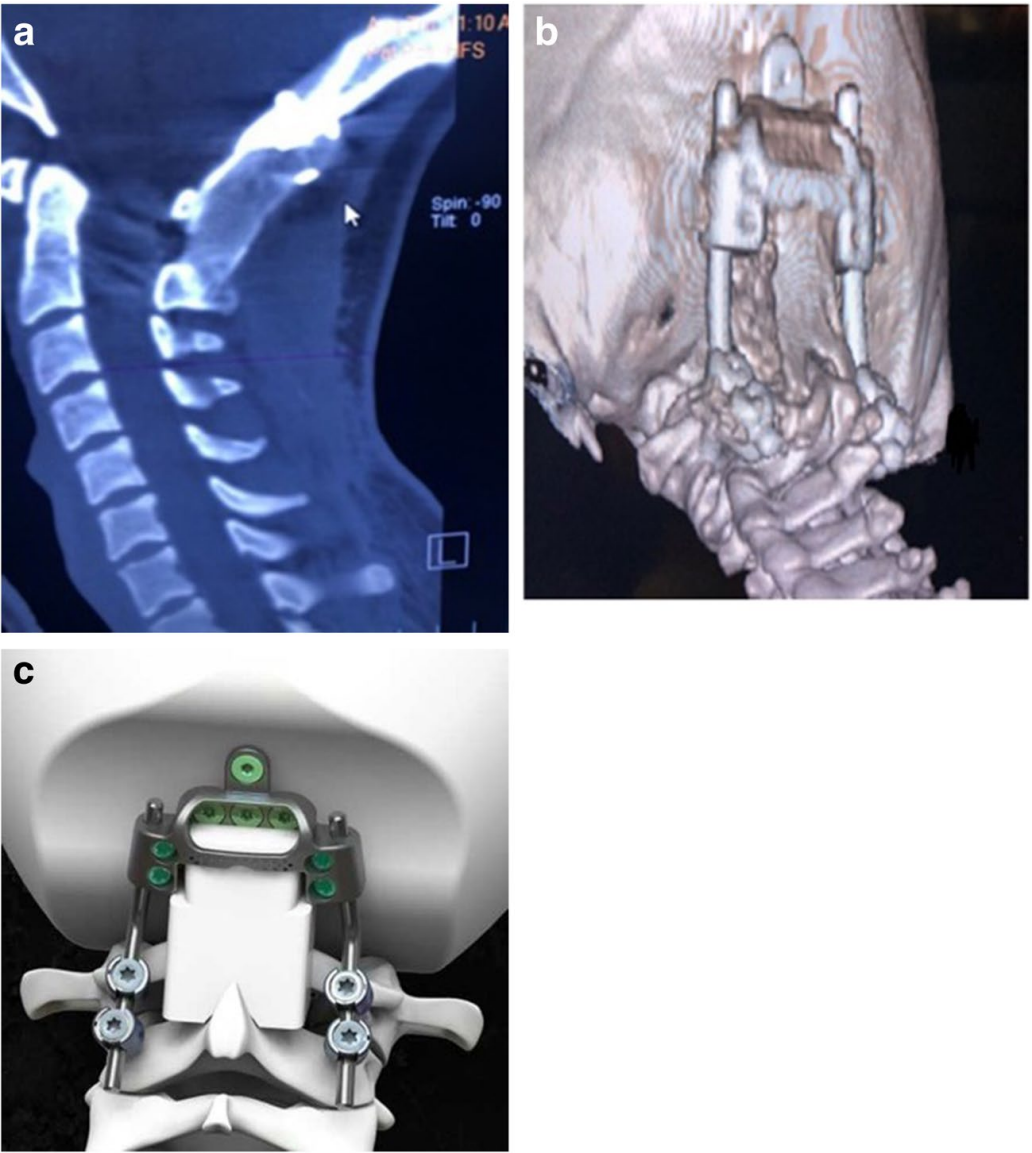
**a** CT scan, mid-sagittal view of the craniocervical junction showing the occipito-cervical fusion/stabilization (OCF). The bone allograft inserts superiorly into the aperture of the suboccipital plate. It lies against the occiput superiorly, the C1 posterior arch, and is notched inferiorly to encompass C2 spinous process and lamina. **b** CT scan, 3D reconstruction, showing the carefully tapered bone graft as it encompasses the occiput superiorly and lamina and C2 spinous process inferiorly. **c** The bone graft is secured within the aperture at the base of the CCI device (Cranio-Cervical Integration device, CCI®, LifeSpine Inc., Huntley, IL). The device has a smooth contour and small footprint to maximize bone surface area for fusion

Patients underwent open reduction/realignment and OCF [13, 14] at a single institution from 2018 to 2020 (Fig. 3a, b).

To the extent possible, intraoperatively we brought the CXA into the normal range (> 140°) and eliminated ventral brainstem compression (pBC2 < 9 mm). We also established a normal or horizontal “gaze angle” (to avoid “star gazing”) and a mandibular axis interval (the measured interval from the anterior aspect of the C2 body to the posterior aspect of the mandible as seen on fluoroscopy or X-ray) > 10 mm and < 24 mm to avoid dysphagia [43]. Bone marrow, aspirated from the iliac crest, was injected into a saline-soaked, tricortical, iliac crest strip allograft for the fusion. The stabilization device used was the Solstice Cranio-Cervical Integration device (CCI®, LifeSpine Inc., Huntley, IL) (Fig. 3c).

The selected device presents a low, smooth profile, and a large aperture to incorporate a large bone graft. Postoperatively, patients were instructed to wear a neck brace for 1 month, and then to begin physical therapy.

Suboccipital decompression was performed for obstruction of CSF flow by Chiari malformation, low-lying cerebellar tonsils, or foramen magnum stenosis (AP diameter ≤ 30 mm). No durotomy was performed. The decompression included the full width of the foramen magnum, 20–25 mm to either side of midline, extending cephalad approximately 12 mm.

Preoperative data were collected from the questionnaires, the history, and neurological exam administered to every patient prior to surgery. Postoperatively, self-report questionnaires were emailed to participants. Additional data were collected from clinic records. Data were managed using Research Electronic Data Capture (REDCap), a secure, web-based software platform designed to support data capture for research studies. Questionnaires were completed by the patients postoperatively at 5–28 months (mean: 15.1 months).

#### Outcome measures

The primary outcome measures were as follows:

- Severity and frequency of head and/or neck pain (both pre-op and post-op pain scores (1–5) as well as questionnaire on post-op improvement in pain severity/frequency. The pain scores were evaluated by comparing those patients with CSF flow obstruction from CMI, LLCT, or FM stenosis against those patients who did not have CMI or LLCT with CSF flow obstruction.
- Use of pain medication

Secondary outcomes were as follows:

- Changes in neurological, autonomic, and connective tissue disorder symptoms
- Functional status (Karnofsky Performance Scale) [44]
- Global Clinical Impression of Change score (changes in activity, symptoms, and quality of life since the surgery or last visit) [45]
- Patient satisfaction survey
- Orthostatic Grading Scale, in which patients reported the frequency and severity of orthostatic symptoms with daily activities before surgery and at final follow-up [46].
- Wood Mental Fatigue Inventory. Before surgery and at the final follow-up, a subset of patients completed this 9-item questionnaire which asks how much in the preceding month the respondent was bothered by difficulty with memory, decision-making ability, concentration, processing, and symptoms of foggy head. Responses include 0 = not bothered at all, 1 = bothered a little, 2 = bothered somewhat, 3 = bothered quite a lot, and 4 = bothered very much; Possible scores ranged from 0 to 36 (maximal mental fatigue) [47, 48].

#### Data analysis

The primary analysis was a descriptive comparison of pre- and postoperative data for surgical patients. Statistical analyses were performed using Stata/IC software, version 15.1 (StataCorp, College Station, TX). Continuous variables are summarized as mean ± standard deviation or median (range), and categorical data were summarized as percentages. Chi-square, Fisher’s exact test, and Student *t*-test were used to analyze categorical and numeric data, respectively. The study was powered at 0.80 for the primary and secondary outcomes of interest, and a conservative two-tailed *p* value ≤ 0.01 was considered statistically significant for this descriptive study.

## Results

Fifty-three patients who had previous OCF met the criteria for inclusion in the study (Table 1).

**Table 1:** Participant demographics

	*N*	%
Sex		
Female	50	94.3
Male	3	5.7
Age (years)		
Median	32	
Range	18–65	
Race		
Asian	2	3.8
Black or African American	2	3.8
White	46	86.8
More than one race	3	5.6
Ethnicity		
Hispanic or Latino	1	1.9
Not Hispanic or Latino	52	98.1
Total	53	

### Precipitating event prior to neurosurgery clinic visit

The mean time between onset of symptoms and neurosurgical evaluation was 12 years. Fifty percent of patients reported the onset of symptoms following a precipitating event; in the remainder, onset was gradual. The most common precipitating events were motor vehicle accidents (*n* = 7), pregnancy and childbirth (*n* = 2), sports injuries (*n* = 3), surgery (*n* = 3, including shoulder, spine, and median arcuate ligament surgery), and infection (*n* = 5).

### Preoperative neurological deficits

The neurological exam was characterized in every patient by a combination of weakness, sensory deficits, loss of the gag reflex, dysdiadochokinesia, hyperreflexia, Romberg sign, Hoffman reflex, absence of abdominal reflex, and abnormal tandem gait. Apart from tussive headache, there were no signal findings that differentiated those patients with Chiari malformation or low-lying cerebellar tonsils from those with findings of instability alone.

### Health care utilization and complications

All 53 patients had OCF and 32 patients also underwent suboccipital decompression in the same surgery. There were no intraoperative complications. Mean hospital length of stay was 4.3 days (SD, 1.2; range 2–8 days). Two to four weeks after surgery, four patients returned for re-operations for wound dehiscence. One of these four patients with a suspected infection returned for a revision of fusion 6 weeks later, after the cultures were negative. Within the follow-up period (average 15 months), 12 patients (23.1%) underwent surgeries for unrelated problems: tethered cord release (*n* = 12), sub-axial fusion (*n* = 3), placement of an intracranial pressure bolt (*n* = 1), and shunt (*n* = 2); and 12 patients (23.1%) were seen in the emergency room for issues not related to the surgery.

### Primary outcomes: headache and/or neck pain and use of pain medication

Postoperatively, there was a significant improvement in headache and neck pain. Headache and neck pain both decreased from very severe, with a mean 4.3/5 pre-op, to moderate, with a mean 3.3/5 post-op (*p* < 0.001). Preoperatively, headache and neck pain scores for subjects with CMI/LLCT and CSF flow obstruction were the same as those with no CMI/LLCT and were similar postoperatively. There was no significant difference between the two groups (see Table 2).

**Table 2:** Average headache and neck pain scores before and after surgery

1 = None, 2 = Mild, 3 = Moderate, 4 = Severe, 5 = Incapacitating	Pre-op headache pain	Post-op headache pain	*p* value	Pre-op neck pain	Post-op neck pain	*p* value
All Subjects* (*n* = 46 headache, *n* = 45 neck pain)	4.3	3.3	< 0.00001	4.3	3.3	< 0.00001
CM/LLCT with CSF flow obstruction (*n* = 28)	4.4	3.2	0.0002	4.3	3.2	0.00002
No CSF flow obstruction (*n* = 18 headache, *n* = 17 neck pain)	4.3	3.5	0.0003	4.3	3.5	0.02

32 subjects had CSF flow obstruction and required a decompression (18 CMI, 13 LLCT, 1 FM stenosis), 3 were excluded because they had been previously decompressed, and 1 was excluded for no post-op pain scores (incomplete postoperative in-office questionnaire)

21 subjects did not have CSF flow obstruction (1 of these had LLCT but no obstruction), 3 were excluded for no post-op pain scores for headache or neck pain, and 1 for no post-op neck pain score. There was no significant difference between the two groups for change in headache (*p* = 0.46) or neck pain (*p* = 0.36).

*Excludes 3 patients who had been previously decompressed for purposes of CMI/non-CMI comparison

**Fig. 4:**
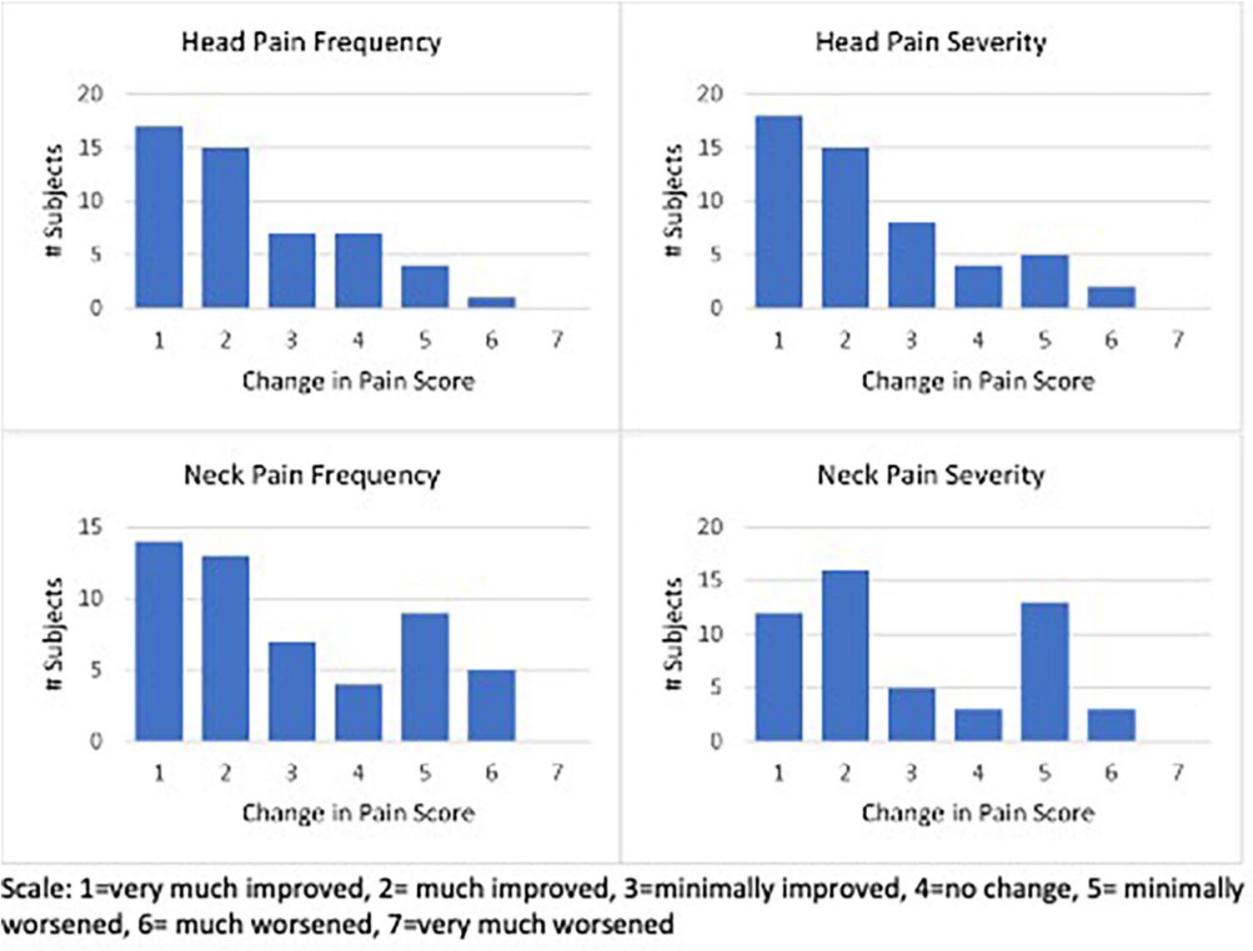
The change in pain score of the head and neck at an average of 15 months (*N* = 52). While there was significant overall improvement in pain frequency and severity of headache and neck pain, some patients (*n* = 15) reported worsened neck pain frequency and severity

Participants were also asked: “Has your head or neck pain changed in severity or frequency?” The patients overall reported significant improvement in terms of severity and frequency of head or neck pain (Fig. 4). There was no difference between the CMI/LLCT group and the non-CMI group of patients: improvement in the CMI/LLCT group was the same as improvement for the non-CMI group. Specifically, there was no significant difference of head and neck pain severity or frequency between the two groups (severity of headache *p* = 0.47; frequency of headache *p* = 0.30; neck pain severity *p* = 0.77, frequency of neck pain *p* = 0.92). Fifty-two percent of patients reported taking less pain medicine.

### Secondary outcomes: improvement of Karnofsky Performance Status and symptoms

Karnofsky Performance Status (KPS) scores improved significantly from a preoperative median KPS = 50 (range 20–80) to postoperative median KPS = 60 (range 40–100) (*p* < 0.0001).

Postoperatively, there were significant improvements in the majority of neurological symptoms: nausea (*p* < 0.001), syncope (*p* < 0.001), presyncope (*p* < 0.001), speech difficulties (*p* = 0.002), concentration (*p* = 0.001), vertigo (*p* = 0.005) and dizziness (*p* = 0.001), photosensitivity and hyperacusis (*p* = 0.002), facial numbness (*p* = 0.002), arm weakness (*p* = 0.002), and incoordination (*p* = 0.001).

There was also significant improvement demonstrated for other important symptoms: fatigue (*p* = 0.001), palpitations (*p* = 0.002), muscle and joint pain (*p* = 0.001), chest pain at rest (*p* = 0.005), shortness of breath at night (*p* = 0.008), abdominal pain (*p* = 0.004), abdominal bloating (*p* = 0.013), pain in legs with ambulation (*p* = 0.013) (Table 3).

**Table 3:** Symptoms of the treated population

Symptoms: mean severity rating 1 = None, 2 = Mild, 3 = Moderate, 4 = Severe, 5 = Incapacitating	Pre-surgery (*n* = 48)	Post-surgery (*n* = 48)	*p* value
*Neurological*			
Hyperacusis/sensitivity to noise	3.2	2.8	0.018
Vertigo	2.5	2.0	0.005
Dizziness/lightheadedness	3.6	2.9	< 0.001
Headache	4.4	3.3	< 0.001
Neck pain	4.3	3.3	< 0.001
Loss of consciousness/syncope	1.9	1.4	< 0.001
Presyncope	3.5	2.7	< 0.001
Concentration difficulties	3.8	3.0	< 0.001
Memory loss	2.9	2.4	0.003
Double vision	2.1	1.5	0.002
Photosensitivity	3.2	2.7	0.011
Facial numbness	2.2	1.7	0.006
Leg weakness	2.8	2.5	0.043
Arm weakness	2.8	2.2	0.002
Nausea/vomiting	3.1	2.5	< 0.001
Poor coordination	3.1	2.5	< 0.001
Speech difficulty	2.3	1.8	0.002
*Constitutional*			
Fatigue	4.3	3.7	0.001
Joint pain	3.9	3.3	< 0.001
*Musculoskeletal*			
Neck pain on bumpy roads	3.8	3.0	< 0.001
Muscle pain at rest	3.5	2.9	< 0.001
Cramps/stiff muscles	3.4	3.0	0.043
Pain in legs while walking	3.1	2.6	0.013
*Cardiovascular/autonomic nervous system*			
Feeling heart beats/palpitations	3.1	2.6	0.002
Chest tightness/pain at rest	2.3	1.8	0.005
Chest pain on exertion	2.5	1.9	0.003
Shortness of breath at night	2.3	1.8	0.008
Shortness of breath at rest	2.2	1.7	0.013
Shortness of breath on exertion	3.2	2.6	0.012
Fingers change color with temperature	2.7	2.4	0.035
Heat intolerance	3.5	2.9	0.018
Elevated temperature of > 101.5°	1.3	1.1	0.044
*Gastrointestinal*			
Abdominal pain	3.0	2.6	0.004
Bloating	2.9	2.6	0.013
Constipation	3.0	2.6	0.011
Heartburn / GERD	2.3	2.0	0.027
Diarrhea	2.2	2.0	0.022
Black stool / blood in stool	1.3	1.1	0.027
*Genitourinary*			
Increased frequency urination	2.66	2.3	0.041
*Psychiatric*			
Anxiety panic	2.6	2.2	0.030

### Patients reported improvement of orthostatic symptoms and mental fatigue

**Fig. 5:**
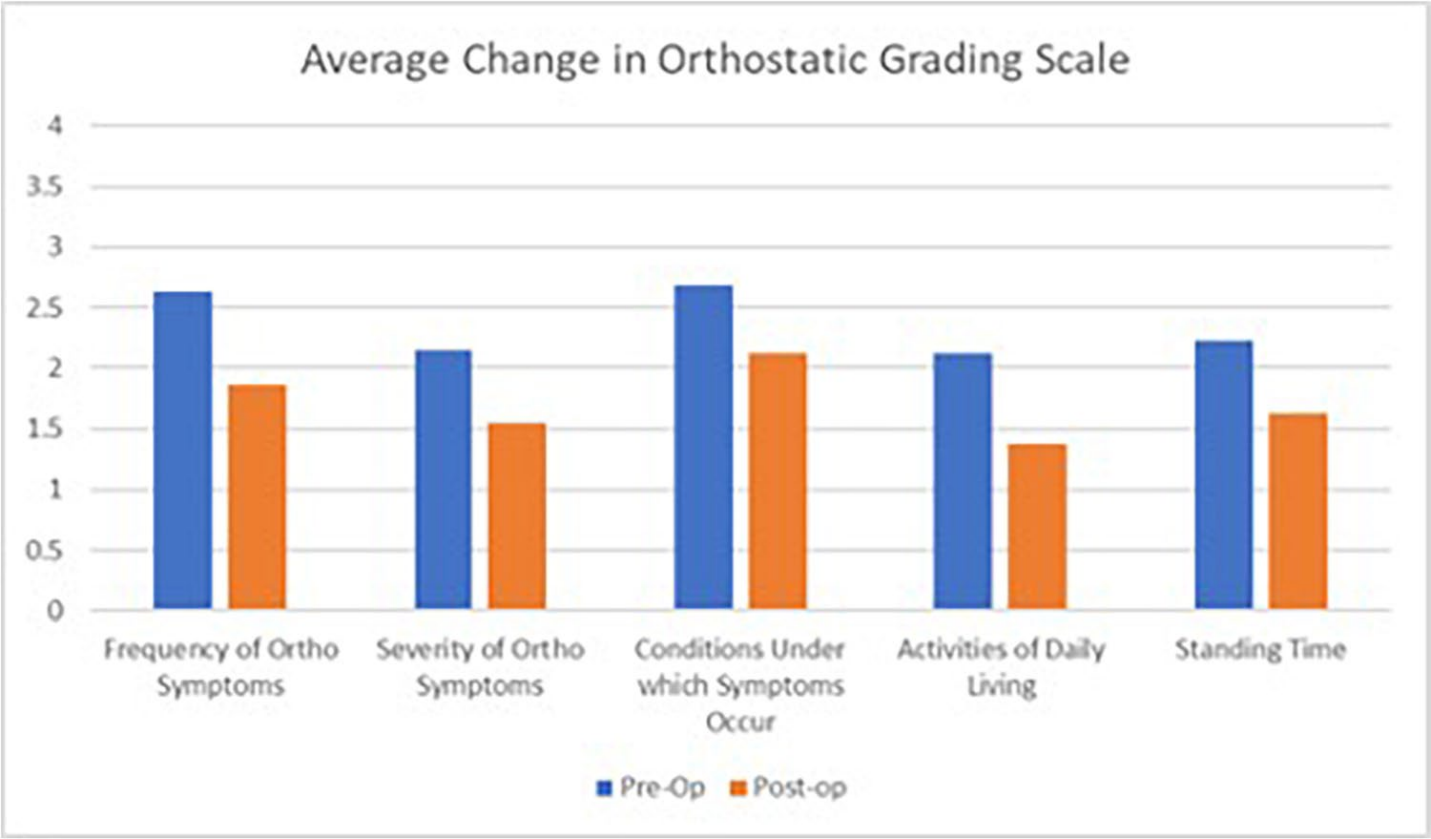
Average change in Orthostatic Grading Scale where 0 = no symptoms, 4 = more severe or frequent symptoms (*n* = 42) *p* =  ≤ 0.006. Patients reported significant improvement of orthostatic symptoms

There was significant improvement in orthostatic symptoms in terms of the frequency, severity, types of activities of daily living, and standing time (*p* = 0.0006) (Fig. 5).

Thirty-two patients completed the Wood Mental Fatigue Inventory (WMFI) before surgery and at the last follow-up. Compared to prior to surgery, these patients reported significant improvement, with less confused or mixed-up thoughts, less difficulty making decisions, greater ability to listen while speaking, less “slow thoughts” and “foggy head” complaints, and less difficulty finding the right words (Table 4). The median WMFI score before surgery was 23 (0 being the best possible score with the least fatigue, and 36 being the maximal score of mental fatigue), and at the latest follow-up, it had improved to 18 (*p* = 0.005).

**Table 4:** Wood mental fatigue

*WMFI measure (n* = *32)*	*Median before surgery*		*Median after surgery*			
Confusion	2	(0–4)	1	(0–3)	NS	
Thoughts mixed up	3	(1–4)	2	(0–4)	*p* < 0.01	
Poor concentration	3	(1–4)	2	(0–4)	*p* < 0.001	
Difficulty making decisions	2	(0–4)	2	(0–4)	*p* < 0.05	
Poor memory recent events	2	(0–4)	2	(0–4)	NS	
Can’t take things in when Speaking	2	(0–4)	2	(0–3)	*p* < 0.001	
Thoughts slow	3	(0–4)	2	(0–4)	*p* < 0.001	
Foggy head	3	(1–4)	2	(0–4)	*p* < 0.001	
Can’t find right words	3	(1–4)	2	(0–4)	*p* < 0.005	
**Total**	23	(0–33)	18	(10–36) 0.005		*p* <

1 no change, 10 worse, 21 improve

0 = not bothered at all, 1 = bothered a little, 2 = bothered somewhat, 3 = bothered quite a lot, 4 = bothered very much

The Wilcoxon Signed Rank Test was used, null hypothesis was rejected at *p* values listed above (NS = null hypothesis was not rejected).

While this study was not designed to assess the impact of Chiari malformation on presenting features or outcome, the authors noted no signal differences in the outcomes of patients diagnosed with craniocervical instability and CMI/LLCT, as compared to those without CMI/LLCT.

### Radiological findings

The radiological findings of CCI are presented (Table 5; *N* = 53).

**Table 5:** Radiological findings

Surgical Case	Harris* (Basion-axis interval) (flex) abnl ≥ 12 mm	Harris Translation (flex-ext) abnl ≥ 4 mm	Grabb-Oakes (mm) abnl ≥ 9 mm	CAA* abnl < 135	Right/Left C1-2 Angular displacement abnl ≥ 41°	CSF flow obstruction	Other radiological findings
1	12	4	1	122*	41/40		
2	11	11		140	46/42	CM1 (5.5 mm)	Poor CSF space maintenance posterior and anterior of the brainstem
3	9	7	7	152	41/42		Did not have adequate flex/ext on MRI; lateral translation/AAI on DMX
4	12	2	9.5	132	37/35		
5	7	7	8	135			Was only able to do 65 total rotations in each direction, insufficient to show any subluxation; lateral translation/AAI on DMX
6	10*	4	7	144*	40/40		AAI, displacement of 41 on another CT, listed on op report
7	12			118	41/40		
8	12	9		139	43/44		On flexion, the length of the brainstem is 3.5 cm. On extension, the length of the brainstem is 2.7 cm
9	12.5		10	128		CM1 (> 5 mm)	
10	10	2		140	40/35		30% facet overlap. On DMX BAI 12 on flexion; significant overhang of the lateral mass of C1 bilaterally; significant change in the para-odontoid space
11	12	6	11		43/44	LLCT	
12	12	1	9	135	41/43	CM1 (6 mm)	Left Kimerle Anomaly
13	12	7.5	9	124			Did not turn far enough for CT; DMX showed increased ADI, significant overhang of the lateral mass of C1 bilaterally; significant change in the para-odontoid space
14	10	6	10	125		LLCT	DMX shows increased ADI, significant overhang of the lateral mass of C1 bilaterally; significant change in the para-odontoid space
15	11.4	2.4	9.5	119	42/42	CM1 (9 mm)	CCI with translation > 4 mm translation
16	10	8	9	127	39/39	CM1 (> 5 mm)	80% loss facet overlap
17	11	8	8	118	40/41	FM stenosis	
18	10	4	11		42/42	LLCT, FM stenosis	80% loss facet overlap
19	10	4		142	39/39	LLCT	80% loss facet overlap
20	15	5	11	120	41/41		congenital non-union of the posterior arch of C1, 90% loss fact overlap. On DMX, significant overhang of the lateral mass of C1 bilaterally; significant change in the para-odontoid space
21	16	6	11	120	43/44		
22	6	0	5.5	136	45/43	CM1 (> 5 mm)	Foramen magnum crowding with CSF obstruction/AAI; 90% loss facet overlap
23	12	4	10	135	35/35		On DMX, significant overhang of the lateral mass of C1 to the left
24	10	4	10	118	40/46		
25	11	5	9	130	43/41		90% loss facet overlap, On DMX significant overhang of the lateral mass of C1 bilaterally; change in the para-odontoid space
26	11	8.5	9	140*	42/-		
27	13		9	128	41/43	LLCT, FM stenosis	On DMX overhang of the lateral mass of C1 bilaterally
28	11.6	2.63	9.5	126	40/42	CM1 (8 mm)	Previous CM decompression
29	11	3	9	118	43/43	LLCT	
30	14	10				LLCT	On DMX increased ADI; overhang of the lateral mass of C1 bilaterally; significant change in the para-odontoid space
31	7	7	7		44/48		Inadequate flexion
32	13	7	10	119			Insufficient rotation, Klippel-Feil C5-C6
33	12	8	10	131	42/42		
34	10	7	10	130	38/40	LLCT, FM narrowing	
35	8	8	8	140*	43/43	CM1 (> 5 mm)	90% loss facet overlap, previous CM decompression
36	9.5	5.5	8	147	45/41		
37	16	5	10	121	43/45	CM1 (14 mm)	Small syrinx
38	11	5	10	133	38/38	LLCT	
39	13	5.5		111	43/42	LLCT	
40	9	5	10	134	44/42	LLCT	
41	12	3.5		134	42/42		Ventral brainstem compression
42	10.5	8.5	9	131	44/45	CM1 (5 mm), FM narrowing	Previously fused C5-C7
43	13	5	11	128	40/49	CM1 (5 mm)	Incompetent alar ligaments with over 4 mm of translation of C1upon C2 on left and right tilt
44	12.5	7.5	10	126	43/44	CM1 (> 5 mm)	
45	12.4	3.4	8.8	128	46/45	CM1 (> 5 mm)	
46	12	2	9	137		CM1 (> 5 mm)	
47	8	3		128		LLCT, FM stenosis	CCI and AAI on DMX (translation > 4mm), significant overhang of the lateral mass of C1 bilaterally
48	13	5		104	43/38	CM1 (> 5 mm)	
49	10	6	11	105		CM1 (20 mm)	Insufficient rotation, previous CM decompression
50	13	5	9.4	127	42/-	LLCT	
51	13*	10.2	3	131*	40/38	LLCT	4 mm lateral translation on DMX
52	9	6	10	121		CM1 (> 5 mm)	Previous CM decompression
53	7	4	9.5	138		CM1 (8 mm)	

*Measured in neutral

Obstruction of CSF flow was assessed in 32/53 (60%) of patients, including 13 with LLCT, 18 Chiari malformation Type 1 (five of whom had previously undergone decompression), and 1 with FM stenosis. One additional patient with LLCT did not have CSF flow obstruction. The assessment of flow obstruction was based upon the limitation of CSF spaces imposed by the Chiari malformation, by a retroflexed odontoid [7], or by foramen magnum stenosis [41]. CSF flow studies were not performed before or after the fusion surgery. As a result of the intraoperative reduction, the preoperative kyphotic CXA (mean 128°) was brought into a normal range (mean postoperative CXA 142.8°; *p* < 0.0001), as measured at 3 months. The preoperative pBC2 (GMO measurement) (mean 9.1 mm) was brought into normal range (mean pBC2 = 6.18 mm; *p* < 0.0001).

### Patient’s satisfaction with surgery and global impression of change

Participants reported a high level of satisfaction with the surgery, and 50/53 (94%) indicated they would repeat the surgery given the same circumstances; three (6%) indicated that they would not repeat the surgery. Forty of the 53 patients (75%) reported global improvement. Nine of the 53 patients (17%) reported worsening of their overall status due to co-morbid conditions, the most prominent of which were severe fatigue, mast cell activation syndrome, POTS, TMJ disorder, jugular vein compression with intracranial hypertension, low pressure syndrome due to presumed CSF leak, dystonia, and the need for further spinal surgery. Indeed, this group of patients reported a mean of 7 co-morbid conditions (Table 6). The Chiari malformation, after suboccipital decompression, was not considered to be a factor in postoperative disability in any of these patients.

**Table 6:** Co-morbid conditions of the patient population (*N* = 53)

Conditions	% Subjects	Conditions (Cont’d)	% Subjects
*Neurospinal/neurovascular*	83% (44)	*Gastrointestinal*	53% (28)
Tethered cord syndrome	49% (26)	Gastroparesis	17% (9)
Chiari malformation	34% (18)	IBS	13% (7)
Intracranial HTN/pseudotumor cerebri	34% (18)	GERD	13% (7)
LLT	26% (14)	Gastritis/colitis/esophagitis	9% (5)
Other (Tarlov cyst, CSF Leak, Lumbar Fusion, Hx TIA/stroke, scoliosis, transverse sinus stent, arachnoid cyst, torticollis, meningocele, Klippel Feil, SI joint dysfunction, Scheuermann’s disease, basilar artery aneurysm, cavernous hemangioma, jugular vein obstruction, spondylolisthesis, subaxial instability)	49% (26)	Other (Gallstones/hx cholecystectomy, SIBO, esophageal dysmotility, diverticulitis, celiacs, chronic constipation, chronic abdominal pain)	28% (15)
		*Musculoskeletal*	43% (23)
		Severe TMJD	26% (14)
*Inflammatory and immune disorders*	74% (39)	Hx joint surgery or bone fracture	17% (9)
		Osteoarthritis	9% (5)
Mast cell disorder/MCAS	45% (24)	Hx trauma	11% (6)
GI (IBS, celiacs, gastritis, colitis, esophagitis)	25% (13)	Other (arthralgia, Ernest Syndrome, muscle spasm or tear)	13% (7)
PANDAS/history of severe infection (e.g. Lyme’s, meningitis)	19% (10)		
		*Cardiac/hematologic*	25% (13)
Asthma	15% (8)	Bleeding disorders (platelet aggregation disorder, prothrombin II def, low fibrinogen)	6% (3)
Allergic rhinitis	13% (7)		
Other (RA, Hla­b27 Positive Arthropathy, CIDP, SLE, MG, Hashimoto’s, Sjogren’s, PI, APS, vitiligo, rosacea)	15% (8)	Other (clotting, APS, Tachycardia, HTN, anemia, diastolic HF, leaky heart valve, aortic root dilation, MVP, hx heart block)	19% (10)
*Neurologic*	98% (52)	*Endocrine*	21% (11)
POTS	58% (31)	Hypothyroidism	11% (6)
Migraine	28% (15)	Other (adrenal insufficiency, Hashimoto’s, Cushing’s, hyperthyroidism, pituitary and thyroid disease, hyperadrenergic state)	13% (7)
Dysautonomia/dystonia	25% (13)		
Occipital neuralgia	23% (12)		
Severe chronic fatigue/CFS	15% (8)	*Psychiatric*	17% (9)
Fibromyalgia	15% (8)	Clinical anxiety	9% (5)
Autonomic neuropathy	11% (6)	Clinical depression	8% (4)
Other neuropathy (peripheral, small fiber)	9% (5)	Other (ADHD, OCD, hx eating disorders,	11% (6)
Other (DSPS, spastic hemiplegia, seizures/epilepsy, CRPS, RSD, PLMD, RLS, hx concussion)	15% (8)	PTSD, cognitive disorder)	
		*Other*	40% (21)
*Genitourinary*	34% (18)	Compression syndromes (TOS, MALS, May-Thurner, SMAS)	15% (8)
Endometriosis	8% (4)		
Kidney or liver dysfunction	8% (4)	Optical disorders	9% (5)
Other (pelvic floor issues, hip dysplasia/impingement, PCOS, interstitial cystitis, hx hysterectomy, uterine fibroids, varicocele)	23% (12)	Integumentary (erythromelalgia, Hailey-Hailey Disease, rosacea, vitiligo, granuloma annulare)	8% (4)
		Sleep apnea/UARS	6% (3)

*ADHD Attention Deficit Hyperactivity Disorder, *APS* Antiphospholipid Antibody Syndrome, *CFS* Chronic Fatigue Syndrome, *CSF* Cerebrospinal Fluid, *CIDP* chronic inflammatory demyelinating polyradiculoneuropathy, *CRPS* Complex Regional Pain Syndrome, *DSPS* Delayed Sleep Phase Syndrome, *GERD* gastroesophageal reflux disease, *GI* gastrointestinal, *HF* heart failure, *HTN* hypertension, *IBS* Irritable Bowel Syndrome, *TIA* transient ischemic attack, *LLT* low lying tonsils, *MALS* median arcuate ligament syndrome, *MCAS* Mast Cell Activation Syndrome, *MG* Myasthenia Gravis, *MVP* Mitral Valve Prolapse, *OCD* Obsessive Compulsive Disorder, *PANDAS* pediatric autoimmune neuropsychiatric disorder, *PCOS* Polycystic Ovary Syndrome, *PI* Primary Immunodeficiency Disorders, *PLMD* Periodic Limb Movement Disorder, *POTS* Postural Orthostatic Tachycardia Syndrome, *PTSD* Post Traumatic Stress Disorder, *RA* Rheumatoid Arthritis, *RLS* Restless Leg Syndrome, *RSD* Reflex Sympathetic Dystrophy, *SI* Sacroiliac, *SIBO* Small Intestine Bacterial Overgrowth, *SLE* systemic lupus erythematosus, *SMAS* Superior Mesenteric Artery Syndrome, *TMJD* Temporomandibular Joint Disorder, *TOS* Thoracic Outlet Syndrome, *UARS* Upper Airway Resistance Syndrome

## Discussion

After failed non-operative management, 53 adult patients with severe head and neck pain, symptoms of the *cervical medullary syndrome*, congruent neurological deficits, and radiological findings of chronic instability of the craniocervical junction (CCI, AAI) underwent open reduction, stabilization, and OCF. Within this cohort of patients with CCI, Chiari Malformation 1 or CSF flow obstruction due to low-lying cerebellar tonsils or foramen magnum stenosis was frequently diagnosed (32/53) and treated with a limited foramen magnum decompression. This outcomes analysis is intended to assess the appropriateness of the indications and the efficacy of OCF in the treatment of instability in these patients (see Surgical Decision Algorithm Supplement). This series should be differentiated from, and not confused with, other series of CMI and basilar invagination [49]. Indeed, the authors concur that OCF is rarely indicated for CMI and should be reserved for patients in whom the primary underlying pathology is mechanical instability and those including the “complex Chiari” in whom significant deformity of the brainstem or upper spinal cord is manifest in the characteristic neurological presentation [13, 21, 28, 39, 40, 50].

### Postoperatively patients reported improvement of pain and neurological symptoms

Postoperatively, most patients reported significant improvement in head and neck pain, in both severity and frequency, and there was a significant measured decrease in use of pain medication. At an average 15 months after surgery, when asked to compare their pain with the preoperative level, 13 patients reported minimal worsening and 3 reported much worsened neck pain. For head pain, 5 were minimally worse, and 2 were much worse. However, a review of the in-office questionnaires before and after surgery (Table 3) of these patients reporting worse pain (Fig. 4) showed that only one had reported increase in headache and only one an increase in neck pain score when compared to pre-op. This discrepancy shows the potential influences of recall bias over time as well as patients’ suffering from comorbid conditions. However, the authors recognize the opportunity to refine the selection criteria for surgery to improve pain outcomes.

The patients with CMI/LLCT were not differentiated from non-CM patients on the basis of pain. There was no significant difference in pain improvement between patients with CMI/LLCT and CSF flow obstruction compared to those without CMI/LLCT.

There was high patient satisfaction following surgery (94%). Patients reported significant objective improvement of syncope and presyncope and in the subjective symptoms of memory and concentration, weakness of the arms, dizziness, vertigo, nausea, speech difficulties, incoordination and balance, fatigue, palpitations, chest pain at rest or with activity, and leg pain while walking. Improvements were also demonstrated for diplopia, leg weakness, Raynaud’s phenomenon (fingers changing color with temperature), urinary frequency, and anxiety, though the latter did not reach statistical significance. The improvement of syncope and presyncope was mirrored in a significant improvement in the frequency and severity of orthostatic symptoms in most types of activities of daily living and in standing time (Fig. 5). Moreover, the improvement of memory, concentration, and fatigue was paralleled in the significant self-reported improvement in terms of the ability to make decisions, with less confused or mixed-up thoughts, greater ability to listen while speaking, less “slow thoughts” and “foggy head” complaints, and less difficulty finding the right words (Table 4).

Improvement of the symptoms of the *cervical medullary syndrome* (alternatively named the *Cervico-cranial syndrome* (ICD 10 code M53.0) or *Brainstem Disability Symptoms* is in keeping with the experience of others describing the treatment of basilar invagination, kyphotic CXA, CCI, and AAI due to incompetence of the craniocervical ligaments [3, 5, 9, 13–17, 22, 26–28, 34, 51].

The improvement of dysautonomia symptoms is attributed to mitigation of deformation of the sympathetic component of the autonomic nervous system [42, 52]. Ventral brainstem compression and instability result in chronic focal encephalopathy, affecting widely collateralized sympathetic neurons in the ventral lateral medulla, which project to preganglionic neurons at multiple spinal levels and also project to “generalist, bulbo-spinal, command neurons” in the central nervous system. The latter influence a broader network and provide tonic drive to cardiac and vascular structures [53, 54]. In this series, the authors attribute significant improvement of autonomic symptoms, in part, to the intraoperative open reduction, and restoration of a stable craniocervical junction with normal ventral brainstem contour.

Notwithstanding the significant improvements of subjective pain and symptoms, the Global Impression of Change found that only 75% of patients reported an improvement in overall quality of life, with 25% of patients reporting no improvement or worsening overall in the follow-up period. The latter must be seen in the context of the many co-morbid conditions from which EDS patients suffer. The legion of conditions (Table 6) included over 115 known diagnoses at the time of surgery of these patients. Commensurate with other reports of EDS patients [16, 19], a high number of patients had been previously treated or were subsequently treated by the authors, for tethered cord syndrome. The authors stress the importance of recognizing the presence of other medical issues, both before and after correction of the CCI, the importance of listening to the patients, and the need for referring them on for further diagnostic evaluation and treatment.

### Radiologic metrics used to assess instability and brainstem deformity

CCI due to ligamentous instability is understandably more common in the populations with HDCT. Ligamentous laxity renders the craniocervical joints ill-equipped to maintain stability with multiaxial movements. Removal of posterior ligamentous and muscular structures in suboccipital decompression for Chiari malformation is associated with a high prevalence of iatrogenic CCI and kyphotic CXA [9, 11, 13–15, 26–28, 50]. The latter appears evident in the EDS population [13, 16, 19, 34, 40, 55].

While the clinical and radiographic algorithms for diagnosis and management of spinal instability in persons with EDS are evolving [56], it is generally recognized that CCI, basilar invagination, and ventral brainstem compression in these patients are often the result of ligamentous incompetence, and that these conditions require dynamic imaging for diagnosis. The authors note increasing acknowledgement of the metrics used in this study.[9, 10, 15, 19, 21, 27,29, 34, 40,49, 55,56,]. The BAI (aka, HHM) and BDI are useful and reliable measures of potentially pathological translation of the basion with respect to the odontoid. These measurements have the advantage that they do not require visualization of the opisthion or the posterior ring of C1, both of which structures are removed with prior suboccipital decompression (Fig. 1) [14, 19, 26, 29, 30, 32, 34, 40, 55, 56].

The mean BAI of 11 mm in our subjects is the same as reported by Marianayagam et al. (2021) among “complex Chiari” subjects who were shown to benefit from OCF [9]. Moreover, the basion-axis interval (BAI) may be measured on mid-sagittal views in flexion and extension to determine whether there is pathological translation [34]. Another important and more recent metric, the condylar-C2 sagittal vertical alignment (C-C2SVA), registers alignment and altered sagittal balance between the cranium (the atlanto-condylar joint) and the axis and is sensitive in the identification of the high-risk Chiari malformation patient that requires occipito-cervical reduction and OCF or ventral brainstem decompression [57].

### Radiological evidence of craniocervical instability is not sufficient to diagnose pathological CCI

Radiological evidence of CCI does not in itself define clinically significant CCI. The authors rely on the doctrine of instability as a condition in which “the loss of the ability of the spine under physiological loads to maintain relationships between the vertebrae, in such a way that there is neither initial damage or subsequent irritation to the spinal cord or nerve roots, and in addition that there is no development of incapacitating deformity or pain due to the structural changes” [58]. Therefore, in the context of HDCT, pathological clinical instability requires the presence of neurological instability as evidenced by pain, symptoms, and deficits referable to the craniocervical junction, in addition to radiological evidence of instability. To be clear, the authors’ decision to consider OCF in patients with EDS was based primarily upon the severity of clinical findings and level of disability. CCI in the EDS populations is usually chronic, associated with a long history of increased pain with excessive motion, and must be diagnosed through the lens of a careful history and neurological examination.

In dealing with the population of patients with EDS, there remains difficulty in the determination of the point at which craniocervical hypermobility becomes CCI [56]. Populations of patients with more ligamentous laxity, such as children and persons with Down syndrome, generally exhibit up to 3 mm of basion-to-axis translation between flexion and extension due to ligamentous laxity [37]. In adults, hitherto, antero-posterior translation > 1 mm at CO/C1 was considered abnormal [13, 26, 29, 32, 34–36, 59]. A more recent retrospective radiology study of 50 adults undergoing upright dynamic MRI demonstrated a mean translation (∆ BAI) of 2.3 mm between flexion and extension. Notwithstanding that the patients of the latter study were imaged for neck pain and may therefore have had some inherent abnormality, the data argue for greater latitude of what constitutes normal translation [60]. We have used antero-posterior translation (∆ BAI) ≥ 4 mm as radiological evidence of instability [14] but acknowledge the need to establish normal parameters of basion-axis translation in patients *without* neck pain, especially in the population with HDCT.

The occipital-atlantal joint is normally a very stable “ball and socket” joint, which permits 10–20° of flexion extension, but less than 1 mm of translation and minimal rotation. This begs the question as to the basis of the pathological atlanto-occipital translation which we, and others, have described above [61]. A recent morphological study compared the occipital-atlantal joints of normal controls (*n* = 80) with patients with Chiari malformation and basilar invagination (*n* = 63). Detailed CT measurements of the occipito-atlantal joints demonstrated significantly smaller condyles and shallower superior facets of the C1 lateral mass in the patients with CMI and basilar invagination; the resulting dysplastic joints were permissive of excessive translation [62].

CCI also results from incompetence of both the condylar–C1 capsular lateral atlanto-occipital and the alar ligaments [7, 8, 13, 14, 40]. In our study, AAI (Fielding Type 1) was present in the majority of patients and was characterized by excessive rotational subluxation or lateral translation, loss of > 80% facet overlap, and decreased spinal canal diameter, but maintenance of a normal atlanto-dental interval [31, 32, 34, 38, 54, 55, 63, 64]. In many cases, the finding of AAI was an important factor in the decision to proceed with the OCF. The argument for AAI as a primary cause of cervical medullary syndrome has been made by Goel [7, 65].

The Park-Reeves consortium found the CXA for subjects needing OCF following posterior fossa decompression was significantly lower (128.8 ± 15.3°) than the subjects who did not require OCF [27]. We agree that correction of the kyphotic CXA (increasing or normalizing the CXA) is associated with improved clinical outcome [9, 13, 14, 21, 26, 27, 39, 50, 52].

### Surgical technique and complications of craniocervical fusion

Open reduction allowed optimization of craniocervical relationships [43]. Suboccipital decompression was performed in 32 patients, in whom there was obstruction of CSF flow. The importance of unimpeded CSF flow through the foramen magnum has been emphasized [41].

Mao et al. noted the difficulty of occipital plate fixation after suboccipital decompression [56]. We were able to position a suboccipital plate following suboccipital craniectomy for Chiari malformation by using a low-profile system. There are many effective craniocervical systems available and many variations in technique, to accomplish the successful alignment and stabilization of the craniocervical junction [40, 66, 67]. We attribute the absence of intraoperative complications and injuries to the vertebral arteries to careful preoperative review of the CT and MRI imaging, precise entry points, angling of the C2 screws, and use of intraoperative fluoro-CT. The low complication rate in the present series is in keeping with others [3, 13, 14, 27, 28, 39, 40]. It is important to emphasize the 20% risk of a high or anomalous vertebral artery foramen, rendering screw placement dangerous [68]. In our series, shorter (16 mm) screws were occasionally placed in those cases where the vertebral artery foramen was very high and medial. Other techniques of stabilization, such as the *occipital condylar screw fixation* and the *inside outside technique*, have demonstrated an excellent record of safety and efficacy [40, 66, 67]. A low complication rate is evident where the OCF surgery is performed regularly, as evidenced by a study of 250 subjects undergoing OCF at one site in which 500 condylar screws were safely placed without screw pullout or vertebral artery impingement [66].

In our study, tricortical iliac crest strip allograft, infused with bone marrow aspirate, supplanted the use of rib autografts [13]. This avoided persistent pain from rib harvest and risk of exacerbating scoliosis. Pain overlying the suboccipital fixation devices, a frequent problem in a previous study, motivated the use in this study of a suboccipital plate with smooth contours, low profile, and small surface area [13]. While the wound dehiscence rate was disappointing, it is a recognized complication of EDS, in which slow wound healing and skin fragility are risk factors. Vicryl may incite inflammation in the epidermis, and consideration should be given to substitution with a non-inflammatory suture material, such as Prolene.

Legitimate concerns exist regarding increased adjacent segment degeneration and the need for further fusions at the subaxial levels [13]. The majority of these patients have significant premature degenerative disc disease and proclivity to subaxial instability [19]. The patient should be cognizant preoperatively of the possibility of needing further cervical fusion. In the authors’ opinion, however, this risk is mitigated by attention to posture and avoidance of injurious activity, especially neck flexion. Moreover, following OCF, the increased neuromuscular control of the neck and ability to exercise and strengthen the neck muscles may serve to improve neck stability.

There are concerns about loss of neck range of motion with OCF. It is the authors’ experience that patients very seldom complain of this, because of the increased range of motion conferred by the HDCT throughout the remainder of the cervical and upper thoracic segments. However, the absence of long-term follow-up of persons undergoing OCF should motivate the utmost care in the selection of patients who are suffering, who meet the indications for surgery, and who have failed a reasonable course of non-operative management.

### Were the surgical indications appropriate?

The study suggests that utilization of the six criteria for OCF was associated postoperatively with statistically significant improvement in pain, mental fatigue, orthostatic and neurological symptoms, as well as non-neurological symptoms, such as fatigue and overall performance of daily activities, as shown by the improvement in KPS. As a retrospective analysis without a control group, this study does not validate the indications proposed for surgery. However, the outcomes analysis does support the *reasonableness* of the surgical criteria used in this study and demonstrates an association of these surgical criteria with favorable outcomes in the majority of cases. Moreover, these surgical criteria are concordant with others discussing OCF in the context of “Complex Chiari” or failed Chiari malformation surgery [3, 5, 9, 12–17, 27, 28, 34, 40].

### Future directions

There remains a lack of consensus as to diagnostic imaging and management algorithms in the CCI and Chiari malformation literature [56, 69]. However, there is an increasing understanding of CCI as the manifestation of underlying ligamentous incompetence. Clearly, there is a need to standardize dynamic studies, to establish normative radiological interpretation of abnormal findings, and to aggregate data for the purpose of developing guidelines to determine which patients are most likely to benefit from surgery for CCI. The development of prospective, multi-center studies to validate the clinical indications and management is strongly recommended.

### Conclusion

CCI is a well-described complication of patients with connective tissue disorders in general and the Ehlers-Danlos syndromes in particular. CCI is often recognized in failed suboccipital decompression for Chiari malformation and in “Complex Chiari.” Individuals with EDS who experience severe headache, neck pain, symptoms of the cervical medullary syndrome, neurological deficits, and radiological findings of CCI and who have failed non-operative management should be considered as potential candidates for OCF. Surgical intervention following utilization of these criteria is associated with significant improvement of pain, neurological symptoms, and disability following open reduction, stabilization, and OCF. However, there remains a need to understand long-term outcomes for this surgery. The many co-morbid conditions observed underscore the severe, multi-organ nature of EDS and the importance of understanding the multi-disciplinary care they require.

## Supplementary information

Below is the link to the electronic supplementary material.

Supplementary file1 (PDF 358 KB)

## Data Availability

Not applicable.
