# Supplementary material for: Craniocervical instability in patients with Ehlers-Danlos syndromes: outcomes analysis following occipito-cervical fusion
Source: Neurosurg Rev. 2024 Jan 2;47(1):27. doi: 10.1007/s10143-023-02249-0 (PMC10758368; doi:10.1007/s10143-023-02249-0)
Supplement: Supplementary file 1 — Supplementary file1 (PDF 358 KB) [file 10143_2023_2249_MOESM1_ESM.pdf]

Surgical Decision Tree: EDS with Suspected Upper Cervical Spine Instability

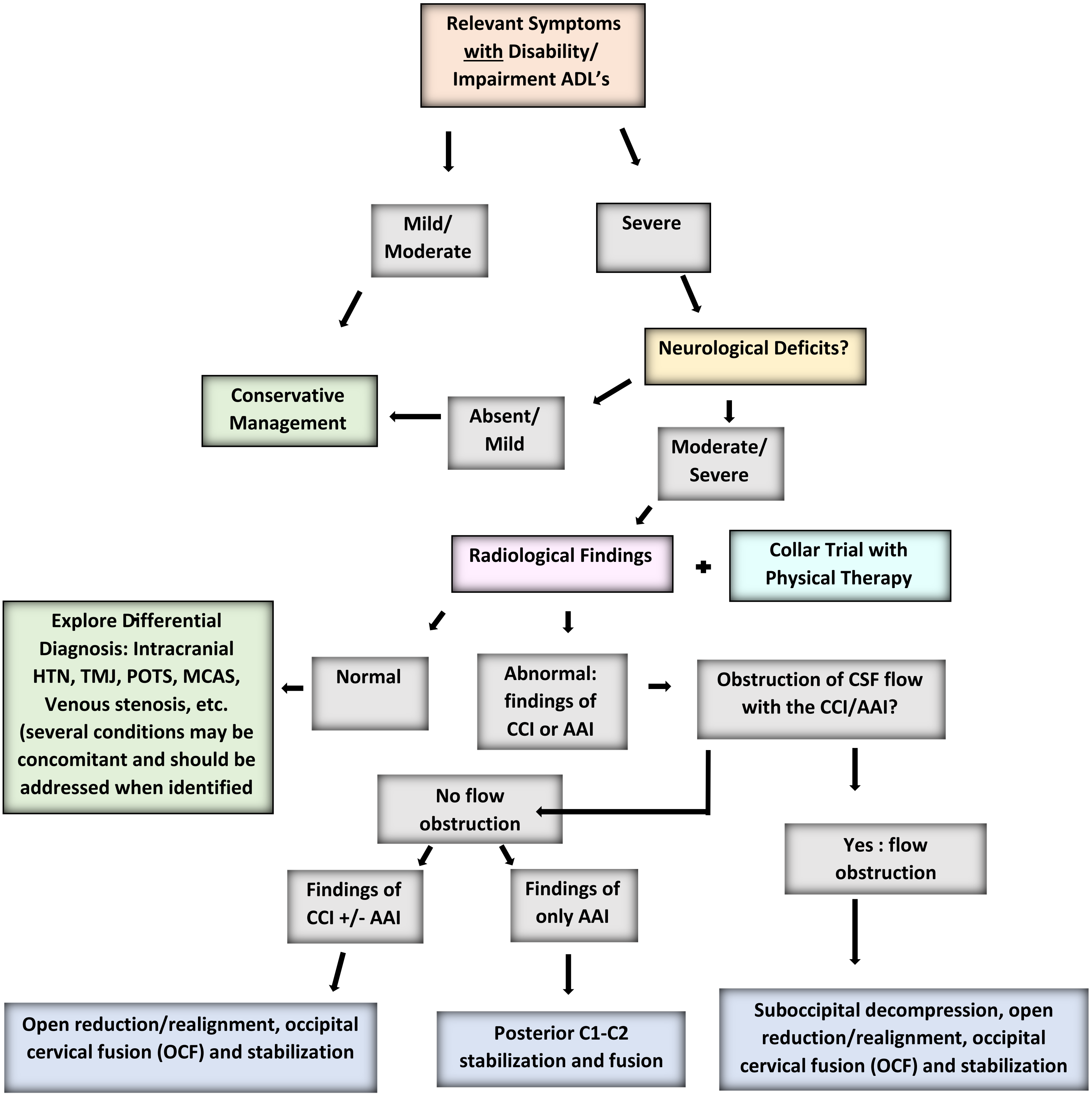

Symptoms

Symptoms of cervicomedullary syndrome : severe headache, neck pain, fatigue, dizziness, vertigo, syncopal episodes, upper and lower extremity weakness and numbness, imbalance, night awakenings, memory difficulties, brain fog, walking and balance problems, sensory changes, visual problems, altered hearing, speech impediments, dysphagia, and micturition issues.

Symptoms of CM1 or Complex Chiari: suboccipital tussive headache exacerbated by Valsalva, cough or straining-dizziness, neck pain, syncope elements of cerebellar dysfunction (dysarthria, incoordination, imbalance, and unsteady gait), lower cranial nerve deficits, hearing and vestibular deficits, Romberg's sign and gait problems. There is often ephemeral facial pain [Milhorat et al., 1999; Tubbs et al., 2011a; Yarbrough et al., 2011]. Brainstem including sleep apnea and dysautonomia, are often found in CM complicated by craniocervical instability or basilar invagination, the "complex Chiari," .

Symptoms of AAI: Headaches (particularly suboccipital or occipital nerve), neck pain, syncope or presyncope, visual changes (e.g. teichopsia, "tunnel" vision, "brownouts" or severe blurring episodes), intermittent dysesthesias of the trunk and extremities, dizziness/lightheadedness, nausea, facial pain, dysphagia, choking, and respiratory issues.

Neurological Deficits

Hypoesthesia to pinprick (over most of cervical, thoracic, lumbar and sacral dermatomes), facial numbness, hyperreflexia (may see hyporeflexia in the context of Vit B12 deficiency), upper or lower extremity weakness, proprioception or vibratory sensation may be decreased, dysdiadochokinesia, Romberg sign, and impairment of tandem gait ,
tenderness over C1/C2 produces pain/nausea/increased neurological deficits

Radiological Findings

Upright, weight bearing MRI with flexion and extension-
CSF flow obstruction: stenosis of foramen magnum , CM1 (≥5mm) or Low Lying Cerebellar Tonsils (3-5mm)
Abnormal findings for CCI:
- **Clivoaxial angle <135°**
- **Horizontal Harris (BAI) ≥12mm or translation ≥4mm** **BpC2/ Grabb Oakes measurement pBC2 ≥9mm**
- **CC2SV measurement >5 mm**

AAI- Retro-odontoid pannus
***Dynamic CT with head rotation 90° to the left and to the right*- Rotation of C1 upon C2 ≥ 41°**
***Flexion/Extension and Rotation must be done sufficiently to ensure accurate readings. If readings are normal, make sure images were taken properly to decide if they may need to be repeated or additional imaging warranted (see below).**

Additional Imaging:

***Digital Motion Xray*- Lateral translation**
significant change in the para-odontoid space
significant overhang of the lateral mass of C1 bilaterally

Collar Trial and Physical Therapy: Collar should improve symptoms (However, the collar may worsen the pain if it presses on C1C2 or upon the mandible in presence of TMJ dysfuntion); PT should be performed under EDS experienced therapist (isometrics, posture training, etc.)
